# Ultrasonic-Assisted Extraction and Antioxidant Potential of Valuable Protein from *Ulva rigida* Macroalgae

**DOI:** 10.3390/life13010086

**Published:** 2022-12-28

**Authors:** Wanida Pan-utai, Thidarat Pantoa, Sittiruk Roytrakul, Jantana Praiboon, Prapat Kosawatpat, Montakan Tamtin, Bussaba Thongdang

**Affiliations:** 1 Department of Applied Microbiology, Institute of Food Research and Product Development, Kasetsart University, Chatuchak, Bangkok 10900, Thailand; 2 Department of Food Chemistry and Physics, Institute of Food Research and Product Development, Kasetsart University, Chatuchak, Bangkok 10900, Thailand; 3 Functional Ingredients and Food Innovation Research Group, National Center for Genetic Engineering and Biotechnology, 113 Thailand Science Park, Phahonyothin Rd., Pathum Thani 12120, Thailand; 4 Department of Fishery Biology, Faculty of Fisheries, Kasetsart University, Bangkok 10900, Thailand; 5 Phetchaburi Coastal Aquaculture Research and Development Center, Coastal Aquaculture Research and Development Division, Department of Fisheries, Phetchaburi 76100, Thailand; 6 Kung Krabaen Bay Royal Development Study Center, Department of Fisheries, Ministry of Agriculture and Cooperatives, Chantha Buri 22120, Thailand

**Keywords:** macroalgae, *Ulva*, protein, ultrasonic-assisted extraction, antioxidant, digestibility

## Abstract

*Ulva* green macroalgae or sea lettuce are rich sources of protein with nutritional benefits that promote health as a future plant-based functional ingredient in the food industry. Alkaline pretreatment improved ultrasonic-assisted protein extraction from *Ulva rigida* biomass. Parameters affecting ultrasonic-assisted extraction of protein were type of solvent, biomass-solvent ratio, biomass preparation and extraction cycle. In vitro digestibility was evaluated from oven- and freeze-dried biomass. Results showed highest concentration and extraction yield of protein from *U. rigida* using alkaline rather than acid and distilled water. A high biomass–solvent ratio at 1:10 or 0.1 g mL^−1^ increased protein extraction. Higher alkaline concentration increased protein extraction. Highest protein extractability was 8.5% dry matter from freeze-dried *U. rigida* biomass, with highest protein extraction and antioxidant activity from extraction of *U. rigida* macroalgae at high alkaline concentrations. *U. rigida* macroalgae oven-dried biomass presented suitable human digestibility. Efficient pretreatment of *U. rigida* maximized protein hydrolysate and bioactive peptide production for wide-ranging applications.

## 1. Introduction

The current trend in the food and biotechnology industries involves replacing goods produced from animals with plant and vegetable components, driven by consumer demand for healthy, wholesome and environmentally friendly products. Consumers regard meals containing algal biomass from seaweed as healthier, and algal production is more environmentally friendly than terrestrial crops. Seaweeds constitute a readily available supply of physiologically active substances, including carbohydrates and proteins, as ingredients for functional nutraceuticals at a cheaper cost than native biomass [1]. Seaweeds are marine macroalgae composed of photosynthetic organisms comprising more than 12,000 species [2]. Seaweeds contain a wide range of bioactive compounds, including polysaccharides, pigments, minerals, vitamins, fatty acids, polyphenols and peptides [3,4], with several advantageous biological traits that promote the creation of functional foods and nutraceuticals. These bioactive substances have been investigated both by in vitro and in vivo model systems for their functional antioxidant, anti-inflammatory, antidiabetic, anticoagulant, antitumor, anticancer, antibacterial and antiviral capabilities [5].

*Ulva rigida* (also known as sea lettuce) is found globally in coastal benthic habitats [6]. The seaweed shows potential for high biomass production and contains high amounts of essential amino acids, approximately 36–42% of total amino acids [7,8]. The amount of protein extracted from macroalgae is affected by factors such as the extraction process, seaweed species and preservation procedure [9]. Bioactive peptides from macroalgae-derived protein have potential health benefits [10]. The initial step in producing bioactive peptides involves locating appropriate protein sources and selection of optimal extraction methods. The structural complexity and hardness of cell wall polysaccharides lead to reduced extraction and use of intracellular bioactive proteins from macroalgae [11], with limited use of seaweed proteins and peptides. Grinding in liquid nitrogen facilitated cell wall disruption, allowing access to seaweed protein. However, this approach is not cost-effective on an industrial scale. High interaction between viscosity and anionic glycoproteins and cell-wall polysaccharides significantly reduces the efficacy of traditional extraction procedures [10].

In a previous study, protein extractions from *U. fenestrate* were obtained using a pH-shift process and mechanical pressing [12]. Proteins were extracted from *Ulva* sp. and *Gracilaria* sp. using various protocols with different reagents and also ultrasonic baths with distilled water [13]. Protein was also extracted from *Ulva* sp. using a high-voltage pulse electric field (PEF) and mechanical press [14]. Ultrasonic-assisted extraction was used for bio substances or phytochemical extraction as a green technology [15]. Ultrasonic pretreatment was applied for protein extraction from *Ascophyllum nodosum* using probe-type ultrasound equipment with 750 W capacity and 20 kHz frequency. This decreased processing time while increasing protein extraction yield [16]. However, significant limitations of ultrasound administered directly to the sample include limited repeatability and possible negative pressure cavitation [17,18]. By contrast, ultrasonic bath systems operated in thermostat mode at 40 kHz are affordable, readily available and capable of treating several samples simultaneously [19]. This study enhanced protein extraction and quality from *U. rigida* biomass using efficient solvents and ultrasonic-assisted mechanical extraction. The essential factors involved in protein extraction from *U. rigida* and antioxidant properties were evaluated. In vitro, gastrointestinal systems were observed from *U. rigida* macroalgae biomass.

## 2. Materials and Methods

### 2.1. Algal Preparation

Sea lettuce as *U. rigida* biomass was obtained from the Phetchaburi Coastal Aquaculture Research and Development Center, Coastal Aquaculture Research and Development Division, Department of Fisheries, Thailand. The biomass was collected after 21 days of cultivation in seawater at salinity 30–32 ppm, washed with clean water to remove any residual culture and subjected to oven- and freeze-drying. Oven-dried biomass preparation involved drying in a hot air oven (model UT6760; Thermo Scientific Heraeus Heating and Drying Ovens, Thermo Fisher Scientific Inc., Thermo Scientific, Dreieich, Germany), with temperature controlled at 60 °C for 3–6 h. Freeze-dried biomass preparation involved freezing at −20 °C for 18–24 h, followed by freeze-drying in a freeze dryer (VFD-12SH; Grisrianthon Co., Klongtoey Bangkok, Thailand) at 30–60 Pa for 20 h. Oven- and freeze-dried samples were milled to 0.5 mm particle size, collected in polyethylene bags and stored in the dark until further extraction and chemical analysis was required.

### 2.2. Protein Extraction Procedure

#### 2.2.1. Solvent Comparison

Preliminary protein extraction from *U. rigida* was performed with different solvents, including acid, alkaline and water. The oven- and freeze-dried biomass of *U. rigida* macroalgae were mixed with 1M HCl (acid), 1 M NaOH (alkaline) and distilled water at biomass–solvent ratios of 1:10 and 1:20. The mixtures were mixed well, followed by ultrasonic-assisted extraction using an electronic ultrasonic bath (DT 100 H, Bandelin, Berlin, Germany) at 35 kHz and 320 W, with extraction time 240 min. The extraction separated the protein by centrifugation at 3461× *g* for 15 min (EBA 200, Hettich, Westphalia, Germany). The same solvent was added to the pellet residues, followed by ultrasonic-assisted extraction for 1–2 cycles. All treatments were performed in triplicate.

#### 2.2.2. Protein Extraction

Protein extraction from green seaweed *U. rigida* macroalgae was evaluated under different conditions of oven- and freeze-dried preparation, alkaline concentration of 0.05–1.0 M and extraction of 1–2 cycles. The biomass–solvent ratio as biomass in g to solvent in ml was performed at 1:10 with ultrasonic-assisted extraction, followed by the optimum condition of protein extraction from algal [20]. Extraction time was 240 min. Protein extraction was separated using centrifugation (DT 100 H, Bandelin, Germany) at 3461× *g* for 15 min. All treatments were carried out in triplicate.

### 2.3. Analytical Methods

#### 2.3.1. Chemical Composition

Oven- and freeze-dried *U. rigida* biomass were determined for biochemical composition following AOAC methods [21]. Moisture content was analysed using oven-drying at under 105 °C to constant weight. Ash content was analysed by igniting the dried samples at 550 °C in an electric furnace. Protein content was analysed by the Kjeldahl method with a factor of 6.25 for nitrogen conversion, while total lipid content was assessed following the modified method of Bligh and Dyer [22]. Briefly, samples were resuspended in a reagent comprising distilled water, methanol and chloroform at a ratio of 0.8:2.0:1.0 and mixed well. The mixture was placed in an ultrasonic bath for 15 min and then centrifuged at 3461× *g* for 15 min for lipid separation. The lipid phase was collected, and the reagent was added to the cell residue debris for repeated extraction until the cells had no colour. The lipid extract was then removed from the contaminated cell debris by filtration through Whatman filter paper GF/C and dried to a constant weight at 80 °C. Crude fibre content was determined using acid and alkaline digestion. The fibre residue was dried to constant weight. The difference in values was used to calculate the carbohydrate content as the sum of moisture, protein, lipid and ash content in 100 g of dry matter. 

#### 2.3.2. Protein Determination

Protein quantification was determined using the Bradford method with a commercial reagent concentrate (Cat No. 500-0006, Bio-Rad Protein Assay Dye Reagent Concentrate, Bio-Rad Laboratories Ltd., Bangkok, Thailand). The quantification measurement was carried out following the manufacturer’s instructions. Briefly, the Coomassie assay reagent was prepared to 5- fold dilution in distilled water. The sample or standard of 40 µL was mixed with 160 µL of reagent and then incubated at room temperature for 5 min. The absorbance was measured at 595 nm by a microplate reader (M965+, Microplate Reader, Metertech, Taiwan). Bovine serum albumin (BSA) was used as the standard. 

Protein extraction yield was calculated by the following equation.

(1) $$Extraction\;yield\;\left({\mathrm{mg}\;g}^{-1}\right)=\;\frac{Protein\times V}{Biomass}$$

where Protein is the concentration of protein measurement (mg mL^−1^), V is the solvent extraction (mL) and Biomass is dried biomass (g).

#### 2.3.3. Free Amino Nitrogen

Determination of free amino acid by the ninhydrin reaction followed Chen et al. [23] with slight modifications. Briefly, a sample or standard of 1 mL was mixed with 2% ninhydrin in 50% ethanol solution and then heated in boiling water for 10 min. The sample was cooled and added with 5 mL of 50% ethanol. The sample was measured at 570 nm absorbance using a UV-vis spectrophotometer (SP-80001, UV/vis Spectrophotometer, Metertech, Taiwan). Glycine was used as the standard.

#### 2.3.4. Total Phenolic Content

The total phenolic content was analysed using the Folin–Ciocalteu colourimetric method with slight modifications [24]. Briefly, a sample or standard of 20 μL was mixed with 0.2 N Folin–Ciocalteu solution (SRL, Maharashtra, India) 100 µL and 0.7 M sodium carbonate solution 80 µL. The mixture was incubated at room temperature for 8 min. Then, distilled water 50 μL was added to the mixture before incubating at 40 °C for 30 min. Gallic acid was used as the standard. The absorbance was measured at 750 nm by a microplate reader (M965+, Microplate Reader, Metertech, Taiwan). Results were expressed as mg gallic acid equivalent (mg GAE·g^−1^).

#### 2.3.5. ABTS Assay

Radical scavenging antioxidant activity was determined according to the ABTS radical scavenging activity following the method of Campos Assumpção de Amarante et al. [25] with slight modifications. Briefly, the solution of ABTS radical was prepared from the reaction between 7 mM ABTS (2,2-azino-bis (3-ethylbenzothiazoline-6-sulphonic acid) diammonium salt) (SRL, India) and 245 mM ammonium persulphate at 505.05 and 5.05 µL, respectively. The ABTS solution was kept at room temperature in the dark for 16 h, and then distilled water was added for dilution to an optical density at 750 nm of 0.7. Then, a sample or standard of 10 μL was mixed with ABTS solution 190 µL. The mixture was incubated for 5 min in the dark. The absorbance was measured at 750 nm by a microplate reader. Ascorbic acid (Sigma-Aldrich, Singapore) was used as the standard. Antioxidant capacity was exhibited as mg ascorbic acid equivalent (mg AAE·g^−1^).

#### 2.3.6. FRAP Assay

The ferric ion-reducing antioxidant power assay was analysed according to Renugadevi et al. [26] with slight modifications. Briefly, the reagent was prepared from 300 mM sodium acetate (pH 3.6) and 10 mM TPTZ (2,4,6-tris (2-pyridyl)-*s*-triazine) (SRL, India) in 40 mM HCl and 20 mM ferric chloride (Sigma-Aldrich, Singapore) at 25, 2.5 and 2.5 mL, respectively. Then, a sample or standard 10 μL was mixed with FRAP reagent 190 µL before being incubated in the dark for 30 min. The absorbance was measured at 593 nm by a microplate reader. Ascorbic acid (Sigma-Aldrich, Singapore) was used as the standard. Results were expressed as mg ascorbic acid equivalent (mg AAE g^−1^).

#### 2.3.7. In Vitro Digestion

The in vitro digestibility of *U. rigida* protein was performed in duplicate by stimulated oral, gastric and duodenal digestion following Smith et al. (2015) [27] with minor modifications.

##### Oral Digestion

Protein digestibility of the sample was studied by mixing with simulated salivary fluid (SSF) containing amylase at 30 units. The mixture was vortexed for 30 s. A portion of the mixture was sampled for use in an oral digestion study. The amylase enzyme was inhibited after 30 s by immersing the mixture in an ice bath for 10 min before storing it in a freezer at −20 °C.

##### Gastric Digestion

Simulated gastric fluid (SGF) containing pepsin was added to the remaining simulated oral digestion mixture and mixed well. The pH was adjusted to 2.5 and the samples were then incubated in a water bath at 37 °C. The samples were taken at 0.3, 11, 22, 33, 44, 55, 66, 77, 77 and 120 min and the enzymatic reaction was stopped by adjusting the pH to 7.5 with 0.5 M NaHCO_3_ and then immersing the samples in an ice bath for 10 min before storing them at −20 °C in a freezer.

##### Intestinal Digestion

Hepatic mix solution (HMS) and pancreatic mix solution (PMS) containing trypsin, alpha-chymotrypsin, pancreatic amylase and lipase was added to the remaining mixture from gastric digesta. The samples were incubated in a water bath at 37 °C. Samples were taken at 0.3, 5, 15, 30, 60 and 120 min and the enzymatic reaction was stopped by adding 0.1 M phenyl methyl sulphonyl fluoride and soaking thee samples in an ice bath for 10 min.

##### SDS-PAGE

SDS-PAGE electrophoresis was performed according to Nisticò et al. (2022) [28] with minor modifications. The polyacrylamide gel was performed in a vertical slab gel apparatus (Mini-PROTEAN system, Bio-Rad, Hercules, CA, USA) using discontinuous gels, which were 1.0 mm thick and contained 0.1% SDS. Equal volumes of C-phycocyanin sample solution and Laemmli sample buffer were mixed and heated at 90 °C for 10 min. A 10 µL of sample mixture and molecular mass standards (MW 10–250 kDa, Bio-Rad, USA) were loaded onto the gel, which contained 15% polyacrylamide slab gel with a stacking gel of 5% polyacrylamide. Samples were separated at room temperature using 80 V for 30 min, followed by 100 V until the end of the run and then visualised by silver staining.

### 2.4. Statistical Analysis

All parameters from the treatments were conducted by one-way analysis of variance (ANOVA) using SPSS (SPPS, Inc., Chicago, IL, USA). Multiple comparisons in all treatments were performed using Duncan’s multiple range test (DMRT) with significance level of 0.05.

## 3. Results

Influences of significant factors on protein extraction from *Ulva rigida* were divided into four sample groups as oven-dried and freeze-dried biomass preparation using various alkaline concentrations (0.05–1.0 M) and 1–2 cycles of extraction. Protein extraction yield, free amino nitrogen, total phenol content and antioxidant activity were evaluated, while protein quality was elucidated using gel electrophoresis after in vitro digestibility.

### 3.1. Chemical Composition

Green seaweed *U. rigida* macroalgae prepared by oven- and freeze-dried techniques were compared, with chemical compositions shown in Table 1. Oven-dried biomass gave higher moisture and lipid contents than freeze-dried biomass, with protein contents similar at 19–22% dry matter. Ash, protein, crude fiber and carbohydrate contents were also similar. Therefore, oven- and freeze-drying methods did not affect cell chemical composition.

### 3.2. Solvent Comparison

**Figure 1:**
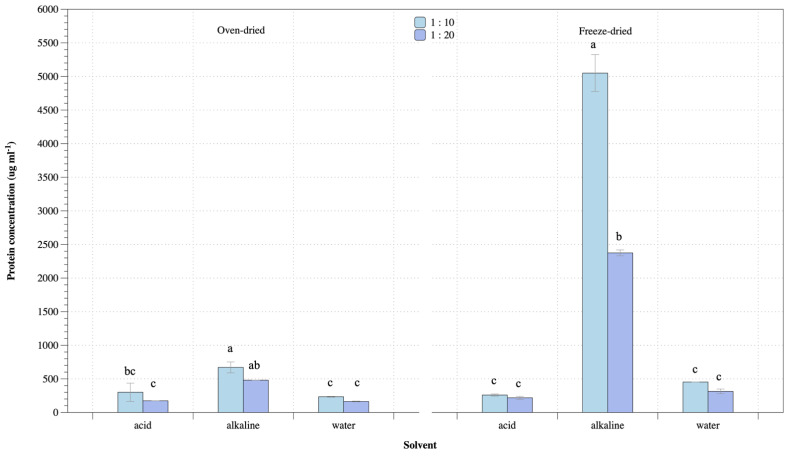
Protein concentration from *U. rigida* using oven- and freeze-dried biomass preparation at different conditions. Data in the same group with different letters are significantly different (*p* < 0.05), including (1) oven-dried biomass and (2) freeze-dried biomass.

Preliminary protein extraction from *U. rigida* was performed using suitable solvents. Figure 1 shows protein concentrations from *U. rigida* using different conditions ranging 163–670 µg mL^−1^ and 218–5,050 µg ml^−1^ for oven- and freeze-dried methods, respectively. Oven-dried biomass gave the highest protein concentration from alkaline extraction but was not significantly higher among different biomass-solvent ratios. Highest protein from oven-dried biomass was 670 µg mL^−1^ obtained from alkaline extraction and biomass–solvent ratio 1:10, while alkaline extraction at 1:10 biomass–solvent ratio of freeze-dried biomass gave highest protein concentration of 5050 µg mL^−1^. Protein concentration from freeze-dried biomass was highest from alkaline extraction, similar to oven-dried biomass preparation. Water and acid solution showed lower protein extraction among all biomass-solvent ratios and biomass preparation. Freeze-dried biomass gave the highest protein concentration at more than 7-fold oven-dried biomass preparation.

**Figure 2:**
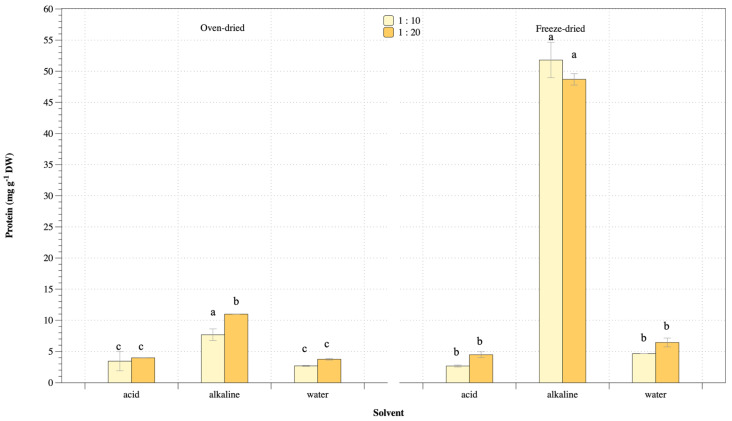
Protein extraction yield from *U. rigida* using oven- and freeze-dried biomass preparation at different conditions. Data in the same group with different letters are significantly different (*p* < 0.05), including (1) oven-dried biomass and (2) freeze-dried biomass.

Protein extraction yields from *U. rigida* macroalgae at various conditions are shown in Figure 2. Results indicated protein yield ranging 2.7–11.0 mg g^−1^ and 2.7–52 mg g^−1^ from oven- and freeze-dried biomass, respectively. Alkaline extraction showed the highest protein yield from both oven- and freeze-dried biomass, whereas water and acid extraction gave low extraction yields. However, results of different biomass-solvent ratios of freeze-dried biomass were not significantly different for protein concentration and extraction yield. Thus, alkaline condition achieved highest protein concentration and extraction yield from *U. rigida* macroalgae.

### 3.3. Protein Extraction

**Figure 3:**
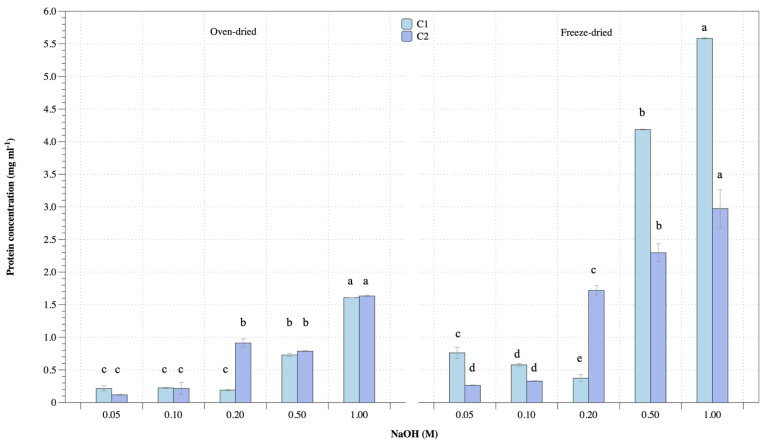
Protein concentration of oven- and freeze-dried *U. rigida* biomass using various conditions. Data were calculated from triplicate treatments with standard deviations. Data in the same group with different letters are significantly different (*p* < 0.05), including (1) oven-dried biomass at cycle I extraction (C1), (2) oven-dried biomass at cycle II extraction (C2), (3) freeze-dried biomass at cycle I extraction (C1) and (4) freeze-dried biomass at cycle II extraction (C2).

**Figure 4:**
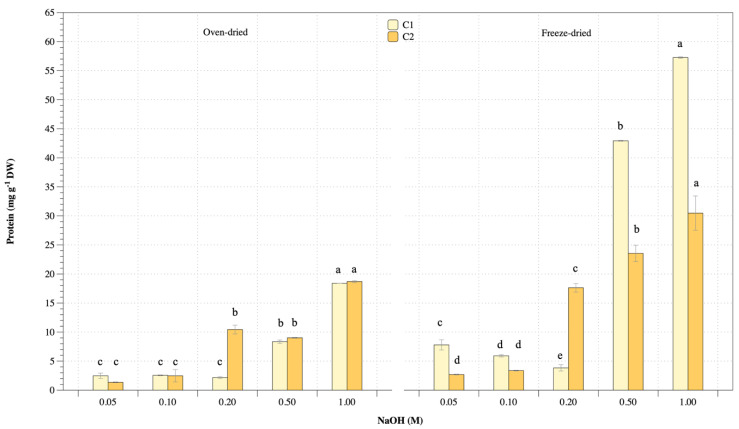
Protein extraction yield from oven- and freeze-dried *U. rigida* biomass using various conditions. Data were calculated from triplicate treatments with standard deviations. Data in the same group with different letters are significantly different (*p* < 0.05), including (1) oven-dried biomass at cycle I extraction (C1), (2) oven-dried biomass at cycle II extraction (C2), (3) freeze-dried biomass at cycle I extraction (C1) and (4) freeze-dried biomass at cycle II extraction (C2).

Significant factors for protein extraction from *U. rigida* macroalgae were performed as oven- and freeze-dried biomass, alkaline concentration and ultrasonic-assisted extraction. Results were divided into four main groups, including oven- and freeze-dried biomass performed under various alkaline concentrations and extraction cycles (cycle 1: C1 and cycle II: C2). Protein concentration and extraction yield are shown in Figure 3 and Figure 4, respectively. Protein concentration of oven- and freeze-dried biomass increased with increasing alkaline concentration (Figure 3). Repeated extraction cycles increased protein release. Oven-dried biomass and cycle I and II extraction gave protein concentrations ranging 0.2–1.6 mg mL^−1^ and 0.1–1.6 mg mL^−1^, respectively, while protein concentrations from freeze-dried biomass and cycle I and II extraction were 0.4–5.6 mg mL^−1^ and 0.3–3 mg ml^−1^, respectively. The highest alkaline concentration gave the highest protein extraction. However, repeated extraction achieved protein release. Freeze-dried biomass showed higher protein concentration than oven-dried biomass. Figure 4 shows protein extraction yield with a similar trend to protein concentration. Protein yields from oven-dried biomass with cycle I and II extraction were 2.5–18 mg g^−1^ and 1.4–19 mg g^−1^ dry matter, respectively, while freeze-dried biomass with cycles I and II gave protein extraction yields ranging 7.8–58 mg g^−1^ and 2.7–30 mg g^−1^ dry matter, respectively. Therefore, extraction and repeated extraction of protein affected protein concentration and yield.

**Figure 5:**
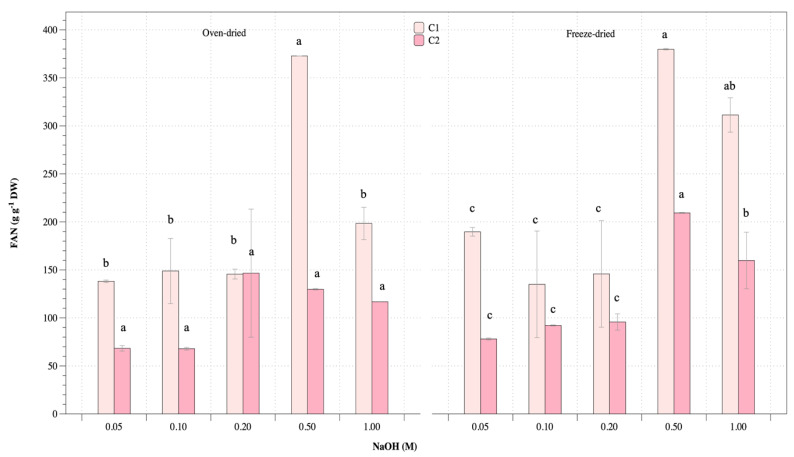
Free amino nitrogen (FAN) of oven- and freeze-dried *U. rigida* extraction using various conditions. Data were calculated from triplicate treatments with standard deviations. Data in the same group with different letters are significantly different (*p* < 0.05), including (1) oven-dried biomass at cycle I extraction (C1), (2) oven-dried biomass at cycle II extraction (C2), (3) freeze-dried biomass at cycle I extraction (C1) and (4) freeze-dried biomass at cycle II extraction (C2).

Free amino nitrogen (FAN) was performed to measure the concentration of free amino acids in protein extraction from *U. rigida* at various conditions, with results shown in Figure 5. Oven-dried biomass for cycles I and II gave FAN values of 139–372 g g^−1^ and 68–147 g g^−1^, respectively, while freeze-dried biomass at cycles I and II gave FAN values of 135–380 g g^−1^ and 78–209 g g^−1^, respectively. Results showed that both oven- and freeze-dried biomass gave higher free amino acids at the first cycle than the second cycle. For both oven- and freeze-dried biomass conditions, highest FAN was obtained from alkaline concentration of 0.5 M.

### 3.4. Antioxidant Capacity

The protein extract seen by the naked eye was as green as bioactive compounds and pigments. Crude protein extract was measured for total phenolic content (TPC) and antioxidant properties by ABTS and FRAP assays under different conditions. Results from oven- and freeze-drying are shown in Table 2 and Table 3. Total phenolic contents from oven-dried biomass under various alkaline concentration at cycles I and II were 0.3–3.7 mg GAE g^−1^ and 0–3.1 mg GAE g^−1^, respectively (Table 3). Highest alkaline concentration from oven- and freeze-dried biomass gave maximum TPC for both cycle I and cycle II extraction. 

Antioxidant capacity was analysed by the ABTS and FRAP assays. ABTS radical scavenging activity was exhibited in protein extracted at different conditions. Results were 2–2.9 mg AAE g^−1^ and 0.5–2.9 mg AAE g^−1^ from oven-dried biomass at cycles I and II, respectively, while ABTS of freeze-dried biomass under various alkaline conditions at cycles I and II ranged 1.6–2.4 mg AAE g^−1^ and 1–2.4 mg AAE g^−1^, respectively. Maximum ABTS radical scavenging activity was achieved from the highest alkaline concentration for freeze-dried biomass preparation. The ferric reducing antioxidant power (FRAP) assay measured protein extracted under different conditions. The results of FRAP at different conditions were 0.1–1 mg AAE g^−1^ and 0.1–1.8 mg AAE g^−1^ from oven- and freeze-dried biomass, respectively. Increasing alkaline concentration showed increased antioxidant activity of FRAP.

## 4. Discussion

Seaweed macroalgae are a good dietary food source as they are simple organisms rich in vitamins, proteins, carbohydrate, trace minerals and other bioactive substances found worldwide [29]. Macroalgae are a viable and affordable biomass source of necessary chemicals with alternative uses in the food, chemical, cosmetic, nutraceutical and pharmaceutical sectors due to their essential biological activity and capacity for biomass production [30,31]. Many *Ulva* sp. or sea lettuce are prevalent in coastal benthic ecosystems [6]. *Ulva* sp. are excellent sources of proteins, pigments, vitamins and minerals and have been studied as nutritional supplements [32]. Proteins are an important class of chemicals that are required for human nutrition. Seaweeds are widely recognised for their nutritional value, particularly in impoverished nations, and macroalgae are a viable alternative nutritious source with high protein content [33]. Protein content varies depending on the phylum [34]. Generally, brown macroalgae have low protein content (3 to 15% dry matter), with green macroalgae (9 to 26% dry matter) and red macroalgae (20 to 47% dry matter) [33,35]. Protein content from *U. rigida* oven- and freeze-dried biomass was obtained at 19–22% dry matter, similar to protein content of green macroalgae. Algae are found in many different and harsh conditions. They contain high concentrations of molecules with various biological functions comprising complex chemical compounds as well as primary and secondary metabolites [36]. Protein content in macroalgae depends on several factors, including geographic region, seasonal cycle and temperature [37,38]. Protein accumulation in cells depends on environmental factors such as cultivation in appropriate salinity and relates to nutrient uptake for metabolism, photosynthesis and growth [39].

Several factors influence protein extraction and various methods promote cellular disruption. The complex hard cell wall is the most influential element in seaweed protein extraction, and its nutrition composition varies between species. Macroalgae proteins are attached to non-protein components such as polyphenols and polysaccharides (agar, alginates and carrageenan), affecting protein extraction efficiency [40,41]. Proteins are generally extracted from macroalgae using aqueous, acidic and alkaline techniques, followed by numerous separation and recovery cycles [16]. Maximum protein extractability was compared between alkaline and ultrasound-assisted extraction. Water is a mild solvent for protein extraction from macroalgae. Seaweeds have various polysaccharides in their cell walls comprising highly integrated networks between enzymes, proteins and biopolymers [11]. Previous reports showed that acid solvent affected protein extraction from brown macroalgae *Ascophyllum nodosum* and *Fucus vesiculosus*, while free amino acids with umami flavor have potential applications in food sectors [42]. By contrast, our results showed low protein extraction under acidic conditions from green *U. rigida* macroalgae. Alkaline extraction gave the highest protein yield because protein solubility depends on pH. Proteins were the least soluble and precipitated out at their isoelectric pH [43]. Alkaline condition generated efficient protein extraction from green *U. rigida* macroalgae.

Fresh macroalgae are seasonal and decay fast after harvesting. To assure year-round availability, they can be maintained via air drying, sun drying, vacuum drying, freezing, or freeze-drying at various temperatures [44]. Drying method also affects the extraction of bioactive substances and cost maintenance. Here, *U. rigida* biomass was prepared using oven- and freeze-drying methods. Although oven drying is less costly, it damages the structure and composition of protein cells, while freeze-drying preserves the bio-substance form of the proteins. In our experiment, oven- and freeze-dried *U. rigida* biomass achieved maximum yield in all protein extractability cycles I and II at 3.7% and 8.5% dry matter, respectively. Freeze-dried biomass preparation results were similar to previous studies. Protein extraction yield from freeze-dried *U. lactuca* biomass was 9.1% dry matter, while other studies reported 10.9% total amino acid extractability [44,45]. Seaweed species type and location impact protein accumulation. Alkaline extraction showed increased protein content. Juul, Danielsen, Nebel, Steinhagen, Bruhn, Jensen, Undeland and Dalsgaard [12] reported that alkaline protein extraction from *U. fenestrate* macroalgae induced cross-linkages and higher pH values gave optimal protein solubility. An adequate quantity of solvent is necessary to dissolve the biomass ingredients and fragmented chemical species to aid in the decomposition of biomass components and conversion to a liquid product [46]. Our results indicated a suitable biomass–solvent ratio of 1:10 for protein extraction from green *U. rigida* macroalgae, comparable to previous studies of the genus *Caulerpa* [47]. The biomass-solvent ratio affects protein extractability [48]. For protein quality, increased solvent volume results in decreased protein concentration, with the optimal ratio dependent on macroalgae species and preparation. Our result of free amino acid concurred with Machado et al. [49], who studied green *U. rigida* macroalgae.

Macroalgae are a rich source of various bioactive compounds with pharmacological and therapeutic applications. Information on bioactive proteins isolated from macroalgae is lacking. An intriguing method for evaluating substances’ safety and potential uses is to measure biological antioxidant activity [50]. Total phenolic content (TPC) and antioxidant capacity (DPPH and FRAP) of green *U. rigida* macroalgae extracted from various methanol concentrations increased with increasing solvent concentration [51]. Our results indicated a similar trend. Oxidation of proteins during processing and storage by reactive oxygen species (ROS) is the principal cause of food degradation that reduces consumer acceptance through quality deterioration and creates hazardous chemicals [52]. Consuming harmful compounds may cause a variety of chronic illnesses in humans, including cancer, arteriosclerosis, aging diabetes, inflammation, coronary heart disease and neurological problems. To prevent food degradation and protect consumers from catastrophic illnesses, lipid peroxidation in food items can be suppressed by applying antioxidant chemicals or preservatives. Antioxidants, as chemical components of biological materials, can extend the shelf life of food by delaying or preventing oxidation. Bioactive peptides are the most prevalent antioxidant compounds found in food [10], and protein extracted from green *U. rigida* macroalgae offers an efficient strategy to improve bioactive peptide applications.

**Figure 6:**
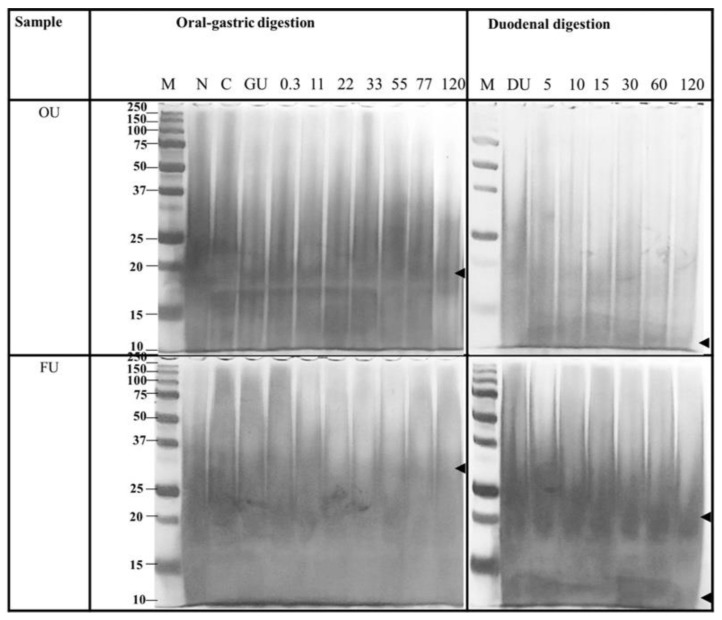
SDS-PAGE profiles of *U. rigida* (OU and FU represent oven- and freeze-dried biomass, respectively) proteins in the oral, gastric and duodenal digestion.

In vitro protein digestibility of oven- (OU) and freeze-dried (FU) *U. rigida* biomass preparation is shown in Figure 6.

Lanes M: molecular weight maker; N: Native protein; C: chew (oral phase); GU: gastric nondigestion (without gastric enzyme); DU: duodenal nondigestion (without duodenal enzyme); 0.3, 11, 22, 33, 44, 55, 66, 77 and 120 in oral-gastric digestion were gastric digested for 0.3, 11, 22, 33, 44, 55, 66, 77 and 120 min, respectively; 5, 10, 15, 30, 60 and 120 in duodenal digestion were duodenal digested for 5, 10, 15, 30, 60 and 120 min, respectively.

Oven- and freeze-dried methods were subject to stimulated oral gastric and duodenal digestion before determining protein digestibility using SDS-PAGE. Results revealed overall polypeptide molecular weight with smearing in the native and in vitro digested SDS-PAGE lenses. The covalent bond between protein and polysaccharides resulted in higher molecular weight than exact proteins [53]. Moreover, the main contained carbohydrates in macroalgae interacted with the proteins, resulting in the overall molecular weight of proteins observed [53]. This study showed low digestibility of protein from *U. rigida*, with high density of SDS-PAGE protein bands left after 120 min of duodenal digestion. This result concurred with Juul et al. (2022) [54], who reported on the limitation *Ulva* protein digestibility in rats. *Ulva* sp. contain high amounts of dietary fibre and minerals, which inhibit protease digestibility [55]. However, higher protein digestibility from *U. rigida* was found in the oven-dried treatment compared with the freeze-dried sample. High-temperature drying treatment eliminated anti-nutrients and increased the accessibility of protease to the *U. rigida* protein substrate.

## 5. Conclusions

Protein was extracted from green macroalgae *Ulva rigida* using chemical and ultrasonic-assisted extraction processes with optimal biomass–solvent ratio of 1:10 for alkaline extraction. Increasing alkaline concentration gave higher protein concentration and yield. Repeated extraction cycles achieved maximum protein. Antioxidant activities were found in protein solutions. The results provide pretreatment information to improve protein hydrolysate and bioactive peptide extraction from green macroalgae. The findings can be used for simple protein extraction or primary pretreatment from algae with reasonably high yield. Our method produced high-value protein components from algae, such as bioactive peptides and functional ingredients, as highlighted by the current protein enrichment techniques employed in various sectors. The use of quality protein from macroalgae in aquaculture, animal feed and human nutrition is expanding.

## Figures and Tables

**Table 1:** Oven-dried biomass (% DW) and freeze-dried biomass (% DW) of *Ulva rigida* preparation.

Chemical Composition	Oven-Dried Biomass	Freeze-Dried Biomass
Moisture (% DW)	7.16 ± 2.64	3.45 ± 0.53
Ash (% DW)	40.21 ± 0.90	45.09 ± 0.69
Protein (% DW)	19.01 ± 0.92	21.93 ± 3.87
Lipid (% DW)	5.67 ± 0.44	0.96 ± 0.54
Crude fiber (% DW)	5.53 ± 0.20	4.04 ± 0.13
Carbohydrate (% DW)	22.42 ± 0.72	24.53 ± 4.24

**Table 2:** Total phenolic content and antioxidant capacity of protein extracted from oven-dried *U. rigida* biomass under different conditions. Data in the same column with different superscripts are significantly different (*p* < 0.05). Data were calculated from triplicate experimental values ± standard deviation (SD).

Alkaline	TPC (mg GAE g^−1^ DW)		ABTS (mg AAE g^−1^ DW)		FRAP (mg AAE g^−1^ DW)	
(M)	Cycle I	Cycle II	Cycle I	Cycle II	Cycle I	Cycle II
0.05	1.03 ^c^ ± 0.03	0.00 ^d^	2.35 ^b^ ± 0.08	0.69 ^b^ ± 0.08	0.09 ^c^ ± 0.01	0.00 ^c^
0.10	0.70 ^d^ ± 0.02	0.00 ^d^	2.25 ^bc^ ± 0.14	0.51 ^c^ ± 0.08	0.09 ^c^ ± 0.01	0.01 ^c^ ± 0.00
0.20	0.33 ^e^ ± 0.03	0.93 ^c^ ± 0.03	2.02 ^d^ ± 0.17	2.88 ^a^ ± 0.01	0.06 ^d^ ± 0.00	0.31 ^b^ ± 0.01
0.50	1.72 ^b^ ± 0.09	1.26 ^b^ ± 0.12	2.86 ^a^ ± 0.01	2.88 ^a^ ± 0.01	0.64 ^b^ ± 0.02	0.32 ^b^ ± 0.01
1.00	3.70 ^a^ ± 0.25	3.06 ^a^ ± 0.02	2.79 ^a^ ± 0.01	2.87 ^a^ ± 0.01	1.02 ^a^ ± 0.00	0.42 ^a^ ± 0.01

**Table 3:** Total phenolic content and antioxidant capacity of protein extracted from freeze-dried *U. rigida* biomass using different conditions. Data in the same column with different superscripts are significantly different (*p* < 0.05). Data were calculated from triplicate experimental values ± standard deviation (SD).

Alkaline	TPC (mg GAE g^−1^ DW)		ABTS (mg AAE g^−1^ DW)		FRAP (mg AAE g^−1^ DW)	
(M)	Cycle I	Cycle II	Cycle I	Cycle II	Cycle I	Cycle II
0.05	1.68 ^c^ ± 0.15	0.44 ^d^ ± 0.02	1.93 ^c^ ± 0.02	1.02 ^b^ ± 0.10	0.17 ^c^ ± 0.01	0.05 ^c^ ± 0.00
0.10	1.50 ^c^ ± 0.11	0.37 ^d^ ± 0.07	1.83 ^d^ ± 0.01	0.94 ^b^ ± 0.03	0.15 ^c^ ± 0.05	0.05 ^c^ ± 0.00
0.20	0.90 ^d^ ± 0.02	1.44 ^c^ ± 0.03	1.60 ^e^ ± 0.01	2.36 ^a^ ± 0.01	0.12 ^c^ ± 0.00	0.26 ^c^ ± 0.06
0.50	3.11 ^b^ ± 0.04	2.28 ^b^ ± 0.06	2.35 ^a^ ± 0.00	2.36 ^a^ ± 0.00	0.97 ^b^ ± 0.01	0.50 ^b^ ± 0.06
1.00	5.19 ^a^ ± 0.06	3.58 ^a^ ± 0.15	2.29 ^b^ ± 0.01	2.32 ^a^ ± 0.01	1.79 ^a^ ± 0.03	0.97 ^a^ ± 0.18

## Data Availability

Not applicable.
